# Purinergic Signalling and Inflammation-Related Diseases

**DOI:** 10.3390/cells11233748

**Published:** 2022-11-24

**Authors:** Tobias Engel, Eva María Jiménez-Mateos, Miguel Diaz-Hernandez

**Affiliations:** 1 Department of Physiology and Medical Physics, Royal College of Surgeons in Ireland, University of Medicine and Health Sciences, D02 YN77 Dublin, Ireland; 2 FutureNeuro, SFI Research Centre for Chronic and Rare Neurological Diseases, RCSI University of Medicine and Health Sciences, D02 YN77 Dublin, Ireland; 3 Discipline of Physiology, Trinity Biomedical Sciences Institute, School of Medicine, Trinity College Dublin, The University of Dublin, D02 R590 Dublin, Ireland; 4 Department of Biochemistry and Molecular Biology, Veterinary School, Complutense University of Madrid, Avda. Puerta de Hierro S/N, 28040 Madrid, Spain; 5 Instituto de Investigación Sanitaria del Hospital Clínico San Carlos, IdISSC, 28040 Madrid, Spain

While acute inflammation is widely accepted as an important response mechanism of cells against tissue injury, sustained inflammatory processes are increasingly recognized as one of the main contributors to numerous diseases, including central-nervous system (CNS)-related and non-CNS-related diseases such as depression, neurodegenerative diseases, type 2 diabetes, hypertension, cardiovascular diseases, chronic kidney disease, osteoporosis, and cancer [1]. Not surprisingly, there is a significant amount of interest in identifying disease-associated inflammatory mechanisms and whether these can be targeted. Moreover, the identification of inflammation-dependent biomarkers may not only support the diagnosis of diseases with an underlying inflammatory condition but also inform the best choice of treatment. 

Extracellular purinergic signalling is mediated via purine nucleosides and nucleotides such as adenosine and adenosine triphosphate (ATP) released into the extracellular space where they activate specific receptors termed purinergic P1 receptors, which respond to nucleosides such as adenosine; and purinergic P2 receptors, which respond to nucleotides such as ATP and uracil triphosphate (UTP). P2 receptors are further subdivided into the fast-acting ionotropic P2X receptors, comprising seven family members, and the metabotropic P2Y receptors, comprising eight family members [2]. Following its release, ATP is rapidly metabolized via ectonucleotidases into different breakdown products (e.g., adenosine diphosphate (ADP) or monophosphate (AMP) and adenosine), signalling molecules in their own right [3]. Compelling evidence over the past few decades has demonstrated that purinergic signalling mediates a broad range of cellular functions in health and disease. Among these, inflammation has attracted the most attention as one of the main pathways by which purinergic signalling contributes to diseases [4]. Much progress has been made in dissecting purinergic signalling cascades and their impact on inflammation, and, most importantly, the use of highly specific drugs targeting different components of the purinergic system has provided compelling evidence for a causal role of purinergic signalling in almost every human pathological condition, ranging from cancer to bone diseases, diabetes, and diseases of the brain [5]. The aim of the present Special Issue is to provide an overview, new evidence of how purinergic signalling regulates inflammatory pathways, and how targeting this signalling system can be used as a new therapeutic strategy for treating diseases associated with inflammation. 

The original research article by Conte et al. investigated the diagnostic potential of the purinergic signalling system for seizures and epilepsy focusing on the ionotropic P2X7 receptor (P2X7R) [6]. Here, the authors show that P2X7R protein levels in plasma are elevated in patients with temporal lobe epilepsy. The authors further show, using P2X7R reporter mice expressing P2X7R fused to the enhanced green fluorescent protein (eGFP), that this increase is most evident in monocytes. Finally, cytokine array analysis in P2X7R-deficient mice identified the cytokine Keratinocyte chemoattractant)/human-growth-regulated oncogene (KC/GRO) as a potential P2X7R-dependent plasma biomarker following status epilepticus and during epilepsy. In a second study, Park et al. [7] showed that the lack of P2X7R led to a decrease in the basal levels of the antioxidant glutathione (GSH) in the mouse hippocampus without altering GSH synthetic enzyme expressions. P2X7R knock-out (KO) increased, however, the expressions of glutamine synthase (GS) and neutral amino acid transporter ASCT2. The authors conclude that P2X7R may be involved in the maintenance of basal GSH levels via the regulation of the glutamate–glutamine cycle and neutral amino acid transports under physiological conditions and suggest that this may constitute a defence mechanism against oxidative stress during P2X7R activation. In a third study, Herman-de Sousa et al. [8] investigated the role of AMP and its metabolite, inosine, in human subcutaneous fibroblasts (HSCF) cell growth and collagen production. Here, the authors show that adenosine, originating from extracellular ATP hydrolysis, favoured normal collagen production by HSCF via A_2A_ receptors. Based on their results, the authors further suggest that inhibition of inosine formation by third-party adenosine deaminase (ADA) cell providers may be a novel therapeutic target to prevent inappropriate dermal remodelling via A3 receptor activation.

In a detailed review by Leavy et al., the impact of inflammation on neonatal seizures was discussed [9]. Of note, targeting the ATP-gated P2X7R has been shown in a previous study by the same group to provide anticonvulsant effects in a model of hypoxia-induced seizures in mouse pups [10]. Finally, a comprehensive review written by Illes et al. [11] discusses how purinergic receptors impact microglia function and how this contributes to disease progression.

In summary, it is increasingly clear that inflammation, in particular sustained inflammation, contributes to disease progression and that the purinergic signalling system represents a possible target to counteract these inflammation-induced detrimental effects.
